# Single Nucleotide Polymorphisms of* CBX4* and* CBX7* Decrease the Risk of Hepatocellular Carcinoma

**DOI:** 10.1155/2019/6436825

**Published:** 2019-05-09

**Authors:** Chao Tan, Chunhua Bei, Xiaonian Zhu, Ying Zhang, Linyuan Qin, Shengkui Tan

**Affiliations:** Department of Epidemiology and Statistics, School of Public Health, Guilin Medical University, Guilin, Guangxi 541004, China

## Abstract

**Background:**

The chromobox (CBX) proteins CBX2, CBX4, CBX6, CBX7, and CBX8, also known as Polycomb (Pc) proteins, are canonical components of the Polycomb repressive complex 1 (PRC1). Abundant evidence indicates that abnormal expression of Pc proteins is associated with a variety of tumors, but their role in the pathogenesis of hepatocellular carcinoma (HCC) has not been fully elucidated. In the present study, we performed a case-control study to investigate the relationship between single nucleotide polymorphisms (SNPs) of* CBX* genes and HCC.

**Methods:**

Nine SNPs on* CBX* genes (rs7217395, rs2036316 of* CBX2*; rs3764374, rs1285251, rs2289728 of* CBX4*; rs7292074 of* CBX6*; and rs710190, rs139394, rs5750753 of* CBX7*) were screened and genotyped using MassARRAY technology in 334 HCC cases and 321 controls. The association between SNPs and their corresponding gene expressions was analyzed through bioinformatics methods using the Ensembl database and Blood eQTL browser online tools.

**Results:**

The results indicated that rs2289728 (G>A) of* CBX4 *(*P* = 0.03, OR = 0.56, 95% CI: 0.33-0.94) and rs139394 (C>A) of* CBX7* (*P* = 0.02, OR = 0.55, 95% CI: 0.33-0.90) decreased the risk of HCC. Interaction between rs2036316 and HBsAg increased the risk of HCC (*P* = 0.02, OR = 6.88, 95% CI: 5.20-9.11), whereas SNP-SNP interaction between rs710190 and rs139394 reduced the risk of HCC (*P* = 0.03, OR = 0.33, 95% CI: 0.12-0.91). Gene expression analyses showed that the rs2289728 A allele and the rs139394 A allele significantly reduced* CBX4* and* CBX7 *expression, respectively.

**Conclusion:**

Our findings suggest that* CBX4* rs2289728 and* CBX7* rs139394 are protective SNPs against HCC. The two SNPs may reduce the risk of HCC while suppressing the expression of* CBX4* and* CBX7*.

## 1. Introduction

The complex pathogenesis of hepatocellular carcinoma (HCC) remains unclear, though it is known that it is influenced by multiple genes and external factors. The main external causes of HCC are the hepatitis B virus (HBV) and the hepatitis C virus (HCV) infection, but the genetic susceptibility to HCC and its mechanism still remains to be discovered. Extensive research on tumorigenesis has led to the discovery of many tumor suppressors and oncogenes, of which overexpression and low expression may lead to cancer. Due to the gene suppressive function of chromobox (CBX) proteins, their abnormities in cancer arouse great attention [1–3].

Human CBX proteins are divided into two main groups: (1) CBX1, CBX3, and CBX5, collectively known as heterochromatin protein 1 (HP1) proteins. They are also known as heterochromatin protein 1*β* (HP1*β*), HP1*γ*, and HP1*α*, respectively. HP1 proteins are critical components in heterochromatin-mediated gene silencing [4, 5]. (2) CBX2, CBX4, CBX6, CBX7, and CBX8, also known as Polycomb (Pc) proteins. They are canonical components of the Polycomb repressive complex 1 (PRC1). Our study focuses on the role of SNPs in genes that encode Pc proteins in the pathogenesis of HCC. PRC1 and PRC2 are two principal multiprotein complexes in Polycomb group (PcG) proteins. PcGs are essential epigenetic regulators that play key roles in cellular development, pluripotency, senescence, and cancer [6, 7].

Emerging evidence from recent studies suggests that CBX proteins are associated with a variety of tumors.* CBX2* inhibition induces cancer cell death, positioning* CBX2* as an attractive drug target for the treatment of advanced prostate cancer [8]. CBX4 is upregulated in breast cancer and exerts oncogenic activities via miR-137-mediated activation of the Notch1 signaling pathway [9]. The expressions of CBX6, CBX7, and CBX8 abnormally alter in glioblastoma multiforme tissues [10]. Overexpression of the* CBX7* gene in hematopoietic stem cells can enhance their self-renewal, giving rise to leukemia [11].* CBX8 *expression is upregulated in colorectal cancer (CRC) cells and clinical samples, and a decrease in CBX8 inhibits CRC cells proliferation [12]. The above-mentioned evidence indicates that the* Pc* gene family is generally upregulated in tumorigenesis. Although other tumor suppressors may also be repressed by the PRC1 complex in the process of tumorigenesis [13, 14], the oncogenic function of* BMI1* and other PRC1 components has been mainly attributed to their repression of the cyclin-dependent kinase inhibitor 2A (*CDKN2A*) locus [15]. Sparmann et al. reported that when another PRC1 canonical component,* BMI1*, was upregulated and accompanied by* MYC*, it caused PRC1 and PRC2 to recruit to the* CDKN2A* locus, resulting in transcriptional repression of the* CDKN2A* locus [16]. The* CDKN2A* locus encodes ARF and INK4A proteins, both of which induce cellular senescence and restrict cell proliferation. When the two proteins decrease, uncontrolled cell proliferation and cancer will occur. Whether abnormal expression of Pc proteins will lead to a similar effect in* BMI1* remains unclear.

The relationship between the* Pc* gene family and HCC is less well-characterized, but there are also some clues in this field. Jie et al. have shown that* CBX4* promotes HCC tumor angiogenesis by governing the HIF-1a protein [17]. Zheng et al. found that the overexpression of* CBX6 *is correlated with tumor progression and poor prognosis in HCC patients [18]. In light of the crucial role of Pc proteins in HCC, mutations of the* Pc* gene family may alter the response of their target genes and cause diseases. However, the relationship between the polymorphisms of the* Pc* gene family and the occurrence of HCC is still poorly understood. Therefore, we conducted a case-control study to explore the association between the SNPs of the* Pc* gene family and the risk of HCC, and to understand the role of the interaction between these SNPs and environmental risk factors such as smoking, drinking, and HBV infection, in the pathogenesis of HCC.

## 2. Methods

### 2.1. Patient Subjects

This study was designed as a hospital-based case-control study. The cases were histologically confirmed as HCC before being obtained from the Affiliated Cancer Hospital of Guangxi Medical University from June 2007 to April 2011. A total of 334 cases were enrolled. The cases were pathologically diagnosed by experienced hepatobiliary surgeons and pathologists according to the* Standard for Diagnosis and Treatment of Primary Liver Cancer* published by the Ministry of Public Health of China. The diagnosed criteria are as follows: tissue samples were collected from puncture biopsies or surgical excisions that were performed on livers exhibiting lesions or extrahepatic metastases. Then, the tissue samples were sent for histopathologic and/or cytological examination. Pathological diagnosis was combined with clinical evidence to comprehensively understand the patients' HBV/HCV infection history, tumor markers, imaging examination, and other information. The enrolled cases did not receive radiotherapy or chemotherapy prior to sample collection. The controls were obtained from the nontumor patients in the Department of Hand Surgery, Spinal Bone Marrow Surgery and Ophthalmology of the First Affiliated Hospital of Guangxi Medical University in the same period as the cases. A total of 321 controls were enrolled. The cases and the controls lived in the same areas (Guangxi, China), and the participants of the two groups were frequently matched according to their age and sex (both* P* >0.05 between two groups, Table 1). All the participants were negative for HCV antibody tests. Before participation, the patient subjects received a detailed description of the study protocol and signed informed consent. The study protocol and the consent forms were approved by the institutional review board of the Tumor Hospital of Guangxi Medical University and the First Affiliated Hospital of Guangxi Medical University.

### 2.2. Sample Collection and Questionnaire Survey

Face-to-face interviews were conducted using an epidemiological questionnaire survey to collect information on the patient subjects. The content of the questionnaire included basic information (such as their name, age, and sex) as well as lifestyle habits that contribute to environmental risk factors (such as smoking habits, alcohol intake, and HBsAg). 2 mL of peripheral blood was collected from each patient subject into a vacuum EDTA anticoagulant tube. The whole genomic DNA was extracted from the blood samples using the phenol-chloroform method and subsequently stored at -80°C.

### 2.3. SNP Screening

The NCBI dbSNP database (https://www.ncbi.nlm.nih.gov/snp/) was used to screen the SNP of* CBX2*,* CBX4*,* CBX6*,* CBX7*, and* CBX8* in the human* CBX* gene family, and the inclusion criterion was MAF>0.05 in the Chinese population (population frequency from the 1000 Genomes Project). Then, the SNPinfo Web Server (https://manticore.niehs.nih.gov/) of the NIEHS database was used to conduct a linkage disequilibrium analysis to distinguish the TAG SNPs from the selected SNPs. It was also used to predict the function of the TAG SNPs. Nine SNPs, namely, rs7217395 and rs2036316 of* CBX2*, rs3764374, rs1285251, and rs2289728 of* CBX4*, rs7292074 of* CBX6*, rs710190, rs139394, and rs5750753 of* CBX7*, were selected for this study. The basic information of the nine SNPs is shown in Table 2. All the SNPs included in the present study were not previously reported in any human diseases. No SNP of* CBX8* fulfilled the inclusion criterion.

### 2.4. Genotyping

MassARRAY system (Agena, Inc., San Diego, CA, USA) was used for genotyping. First, the target fragments containing SNPs to be detected were amplified from the samples by PCR reactions. After which, the PCR products were treated with shrimp alkaline phosphatase (SAP, Agena, Inc.) to remove the free dNTPs from the reaction system. Subsequently, single base extension reactions were carried out and purified using resin. The purified products were then added to 384-well SpectroCHIP bioarray chips and tested using a MALDI-TOF mass spectrometer (MassARRAY Analyzer 4.0, Agena, Inc.).

### 2.5. Statistical Analyses

EpiData3.1 software (downloaded from http://www.epidata.dk/links.htm, EpiData Association, Denmark) was used for data entry and consistency check. The SPSS 19.0 software (IBM, Corp., Armonk, NY) was used for statistical analyses. The quantitative data and categorical data were analyzed using *t*-test and *χ*^2^ test, respectively. The logistic regression model was used for calculating the odds ratio (OR), 95% confidence interval (CI) of OR, SNP-environmental factors interaction, and SNP-SNP interaction. Linear regression analyses were used to test the correlations between the SNPs and the expression levels of their corresponding genes. The size of the tests is *α* = 0.05. False discovery rates (FDRs) were calculated using the R software (Version 3.2.2) following the Benjamini & Hochberg Procedure. The gene expression data was obtained from the HapMap 3 database (https://www.sanger.ac.uk/resources/downloads/human/hapmap3.html), and the data was collected from experimental detection on 76 lymphoblastoid cell lines derived from the CHB (Chinese Han in Beijing, China) population. Gene expression data was downloaded from the submissions of Kolesnikov. et al. [19] in Functional Genomics Data (http://www.ebi.ac.uk/arrayexpress), and the genotype data was downloaded from the Ensembl database (http://www.ensembl.org). Furthermore, the data of the relationship between the SNPs in this study and their gene expression was searched using the Blood expression quantitative trait loci (eQTL) browser (http://www.genenetwork.nl/bloodeqtlbrowser/) [20].

## 3. Results

### 3.1. General Demographic Characteristics of Patients

No statistically significant difference was found in age and sex between the cases and the controls (*P*>0.05), but their smoking habits, alcohol intake, and HBsAg were statistically different in the two groups (*P*<0.05), as presented in Table 1. The genotype frequencies in the controls of all 9 SNPs were in line with the Hardy Weinberg equilibrium (HWE), as shown in Table 2.

### 3.2. Relationships between CBX SNPs and HCC

The adjusted values of their age, gender, smoking habits, alcohol intake, and HBsAg after logistic regression analyses showed that both the GA genotype of rs2289728 (*P* = 0.03, OR = 0.56, 95% CI: 0.33-0.94) and the CA genotype of rs139394 (*P* = 0.02, OR = 0.55, 95% CI: 0.33-0.90) reduced the risk of HCC. No statistically significant association was found between other SNPs and the risk of HCC (Table 3).

### 3.3. Gene-Environment and SNP-SNP Interaction

Gene-environment and SNP-SNP interaction analyses based on the two positive loci (rs2289728 and rs139394) were conducted. No interaction between the two loci and environmental risk factors was found, as shown in Table 4. SNP-SNP interaction between rs710190 and rs139394 reduced the risk of HCC (*P* = 0.03, OR = 0.33, 95% CI: 0.12-0.91), as shown in Table 5.

### 3.4. Correlation between the SNPs of CBX and the Expression of Their Corresponding Genes

**Figure 1:**
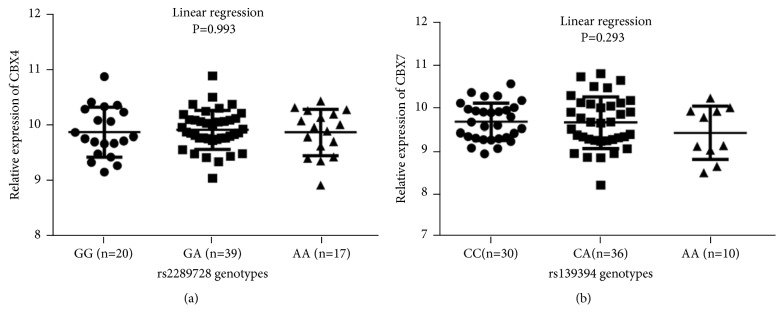
Correlation between the identified SNPs and the expression of their corresponding genes in 76 HapMap CHB lymphoblastoid cell lines. (a) Linear regression analyses of the correlation between rs2289728 genotypes and the expression of CBX4. (b) Linear regression analyses of the correlation between rs139394 genotypes and the expression of CBX7.

The results of eQTL analyses showed that rs2289728 and rs139394 had no effect on the expression of their corresponding genes in the CHB population (*P*>0.05, Figure 1). Taking into consideration that the CHB population gene expression data in the HapMap 3 database had a small sample size (n = 76), we did further research on the gene expression data in the interracial eQTL database (Blood eQTL) and found that the rs2289728 A allele significantly reduced the expression of* CBX4* (*P* = 1.84E-06), and the rs139394 A allele significantly decreased the expression of* CBX7* (*P* = 3.49E-09).

## 4. Discussion

In this study, we revealed the association between the* CBX* gene family SNPs and the risk of HCC through a case-control study. We found that both the independent and combined effects of* CBX4 *rs2289728 and* CBX7 *rs139394 reduced the risk of HCC. Analyses of the eQTL data indicated that rs2289728 and rs139394 suppressed the expression of* CBX4 *and* CBX7*, respectively. Our preliminary findings demonstrated the role of* CBX4* and* CBX7 *in the pathogenesis of HCC and provided a new way to elucidate the molecular mechanisms underlying the pathogenesis of HCC.

PRC1 and PRC2 work together to take part in the target gene transcriptional repression activities of PcG. Furthermore, both PRC1 and PRC2 can suppress the expression of their target genes independently [21]. Target genes transcriptional repression effects of PRC1 were mainly attributed to histone H2A ubiquitination interference with transcription elongation by RNA polymerase II. The H2A ubiquitination activity is then mediated by E3 ubiquitin-protein ligases RING1 or RING2 components of PRC1 [22]. Pc proteins (CBX2, CBX4, CBX6, CBX7, and CBX8) serve as canonical components of PRC1 complexes to suppress the transcription of target genes. As mentioned above, overexpression of PRC1 components can lead to the abnormal repression of the* CDKN2A* locus (*Ink4a*/*Arf *locus), which encodes two tumor suppressing proteins, ARF and INK4A. As a result, uncontrolled cell proliferation and tumorigenesis will occur. Nevertheless, although overexpression of other PRC1 components such as* BMI1* [16],* EZH2*, and* SUZ12* has been demonstrated to be correlated with cancer, the role the Pc gene family plays in cancer remains poorly understood [23].* CBX4* is generally identified as an oncogene in HCC. A decrease in* CBX4* leads to decreased cell proliferation and slower cell cycle progression in HCC cells [24]. On the other hand, overexpression of CBX4 increases the proliferative, invasive, and migratory capacities of the HCC cell line HepG2 [25].* CBX4 *enhances hypoxia-induced vascular endothelial growth factor (VEGF) expression and angiogenesis in HCC cells [17]. The data reported here proposes that* CBX4 *rs2289728 decreases the risk of HCC by repressing the expression of* CBX4*. Our finding is in line with the view that* CBX4* is an oncogene, and the mechanism behind the decreased risk of HCC by rs2289728 might be that the SNP relieves the inhibition on* CDKN2A* locus by suppressing* CBX4*.

Whether* CBX7 *is an oncogene or a tumor suppressor still remains controversial. It is likely that the role of CBX7 in cancer is diverse, depending on the type of tissue. For instance,* CBX7* is upregulated in follicular lymphoma and prostate cancer. The oncogenic gene characteristics of* CBX7* are attributed to its direct repression on the* CDKN2A* locus [26, 27], which is consistent with the function of tumorigenesis of some PRC1 components such as* BMI1*,* EZH2*, and* SUZ12*. On the contrary, Forzati et al. found that decreased levels of* CBX7* caused mice to develop liver and lung tumors, accompanied by an overexpression of cyclin E and* CCNE1*. Moreover,* CBX7 *was found to be significantly downregulated in human lung carcinoma tissues, which suggests that* CBX7 *functions as a tumor suppressor in these types of tissue by repressing cyclin E and* CCNE1* [28]. We found that* CBX7 *rs139394 reduced the risk of HCC by suppressing the expression of* CBX7*, which does not concur with the results of Forzati et al. However, the finding is in line with the concept that* CBX7 *is an oncogene, which might imply that* CBX7* functions differently in mice than in humans.

In addition to genetic susceptibility, environmental risk factors also play an important role in HCC pathogenesis. The independent role of genes and environmental risk factors in HCC and their combined effects has been previously proven [29–32]. Although our results indicated that no gene-environment interaction was found, we observed that SNP-SNP interaction between rs710190 and rs139394 decreases the risk of HCC. These findings suggest that rs710190 is neither an independent risk factor nor a protective factor of HCC, but the combined effect of the interplay between SNPs can result in the alteration of the genetic susceptibility to HCC.

However, the present study has some limitations: (1) The study has a small sample size (334 cases and 321 controls). Nevertheless, our samples were obtained from Guangxi, China, which is an area with high HCC incidence. Additionally, loci with high frequencies of mutation within the Chinese population were selected. As a result, we could achieve an appropriate statistical power to discover two positive SNPs. (2) Instead of using in vitro or in vivo experiments, we used bioinformatics methods to validate the relationship between the two positive SNPs and the expression of their target genes. Hence, several improvements that can be made to this study are to expand the sample size and to conduct cell and animal experiments to explore the roles of* CB4* and* CBX7* polymorphisms in HCC.

## 5. Conclusions

The results presented in this study suggest that two SNPs of the* CBX *family:* CBX4* rs2289728 and* CBX7* rs139394 decrease the risk of HCC. A possible mechanism may be that the two SNPs downregulate the expression of* CBX4 *and* CBX7*, respectively, leading to an increase in* CDKN2A* locus expression. Further intensive investigation needs be recruited to understand the molecular mechanism underlying our findings.

## Figures and Tables

**Table 1:** Distributions of general demographic characteristics and environmental risk factors in cases and controls.

Characteristics	Cases (n = 334)	%	Controls (n = 321)	%	*P*-value
Age (years)^a^	49.1 ± 10.87	-	49.31 ± 12.06	-	0.82
Gender					0.25
Male	279	83.53	257	80.06	
Female	55	16.47	64	19.94	
Smoking habit					**0.001**
Yes	120	35.93	36	11.21	
No	214	64.07	285	88.79	
Alcohol intake					**0.001**
Yes	116	34.73	33	10.28	
No	218	65.27	288	89.72	
HBsAg					**0.001**
Positive	273	81.74	37	11.53	
Negative	61	18.26	284	88.47	
Family history^b^					**0.001**
Yes	85	25.45	3	0.94	
No	249	74.55	318	99.06	

Bold values indicate statistical significance. a: the ages of the patient subjects were represented as Mean ± SD. b: since the positive rate in the control group was extremely low, we did not include this factor in the logistic regression models in order to avoid causing model bias.

**Table 2:** The results of HWE tests in controls and the basic information of SNPs.

SNPs	*χ* ^2^	*P*-value	Wild/Mutant Allele	Location	MAF in Chinese population (1000 Genomes Project)
rs7217395	0.96	0.33	C>T	chr17:79780770	0.38
rs2036316	2.65	0.10	C>T	chr17:79780979	0.37
rs3764374	0.09	0.76	C>T	chr17:79841497	0.12
rs1285251	2.36	0.12	C>T	chr17:79836024	0.33
rs2289728	0.01	0.91	G>A	chr17:79838055	0.48
rs7292074	0.79	0.37	A>C	chr22:38870446	0.47
rs710190	0.15	0.70	T>C	chr22:39131785	0.23
rs139394	0.15	0.70	C>A	chr22:39142209	0.35
rs5750753	1.56	0.21	C>G	chr22:39132501	0.41

**Table 3:** Associations between SNPs and HCC.

Genotypes	Cases [n (%)]		Controls [n (%)]		*OR* (95% *CI*)^a^	*P*-value ^a^	FDR
	n	%	n	%			
rs7217395							
CC	154	46.11	138	42.99	1	-	-
CT	129	38.62	137	42.68	0.97 (0.60-1.58)	0.90	0.90
TT	33	9.88	26	8.10	1.41 (0.63-3.16	0.40	0.60
CT/TT	162	48.50	163	50.78	1.04 (0.66-1.65)	0.87	0.89
Genotyping failed	18	5.39	20	6.23	-	-	
rs2036316							
CC	95	28.44	94	29.28	1	-	
CT	136	40.72	132	41.12	0.91 (0.52-1.57)	0.72	0.90
TT	86	25.75	68	21.18	1.30 (0.70-2.42)	0.40	0.60
CT/TT	222	66.47	200	62.31	1.04 (0.63-1.72)	0.89	0.89
Genotyping failed	17	5.09	27	8.41	-	-	
rs3764374							
CC	214	64.07	161	50.16	1	-	
CT	101	30.24	100	31.15	1.24 (0.75-2.04)	0.40	0.72
TT	16	4.79	14	4.36	0.78 (0.27-2.23)	0.64	0.82
CT/TT	117	35.03	114	35.51	1.16 (0.72-1.87)	0.53	0.80
Genotyping failed	3	0.90	46	14.33	-	-	
rs1285251							
CC	165	49.40	152	47.35	1	-	
CT	120	35.93	132	41.12	1.16 (0.72-1.88)	0.55	0.83
TT	43	12.87	18	5.61	2.260 (0.99-5.16)	0.05	0.45
CT/TT	163	48.80	150	46.73	1.31 (0.83-2.08)	0.24	0.43
Genotyping failed	6	1.80	19	5.92	-	-	
rs2289728							
GG	116	34.73	88	27.41	1	-	
GA	146	43.71	152	47.35	**0.56 (0.33-0.94)**	**0.03**	0.14
AA	66	19.76	64	19.94	0.63 (0.33-1.22)	0.17	0.51
GA/AA	212	63.47	216	67.29	**0.58 (0.35-0.94)**	**0.03**	0.23
Genotyping failed	6	1.80	17	5.30	-	-	
rs7292074							
AA	120	35.93	135	42.06	1	-	
CA	152	45.51	143	44.55	0.52 (0.25-1.07)	0.08	0.24
CC	54	16.17	30	9.35	0.67 (0.33-1.36)	0.26	0.59
CA/CC	206	61.68	173	53.89	1.41 (0.89-0.24	0.15	0.34
Genotyping failed	8	2.40	13	4.05	-	-	
rs710190							
TT	199	59.58	176	54.83	1	-	
CT	113	33.83	106	33.02	1.05 (0.65-1.71)	0.84	0.90
CC	17	5.09	18	5.61	1.04 (0.38-2.88)	0.94	0.94
CT/CC	130	38.92	124	38.63	1.05 (0.66-1.67)	0.84	0.89
Genotyping failed	5	1.50	21	6.54	-	-	
rs139394							
CC	186	55.69	147	45.79	1	-	
CA	106	31.74	128	39.88	**0.55 (0.33-0.90)**	**0.02**	0.14
AA	35	10.48	25	7.88	1.07 (0.47-2.43)	0.87	0.94
CA/AA	141	42.22	153	47.66	**0.63 (0.39-0.99)**	**0.05**	0.23
Genotyping failed	7	2.10	21	6.54	-	-	
rs5750753							
CC	82	24.55	99	30.84	1	-	
CG	158	47.31	141	43.93	1.54 (0.90-2.62)	0.12	0.27
GG	86	25.75	67	20.87	1.57 (0.83-2.96)	0.17	0.51
CG/GG	244	73.05	208	64.80	1.55 (0.93-2.56)	0.09	0.27
Genotyping failed	8	2.40	14	4.36	-	-	

Bold values indicate statistical significance.

a: adjusted age, gender, smoking habits, alcohol intake, and HBsAg values after logistic regression. Values of these covariates in logistic regression models were shown in Table S1.

**Table 4:** Results of gene-environment interaction analyses.

Factors	*β*	SE (*β*i)	*OR* (95% *CI*)^a^	*P-*value ^a^
rs2289728×Smoking habit	0.27	0.66	1.31 (0.36-4.76)	0.68
rs2289728×Alcohol intake	-0.42	0.68	0.66 (0.17-2.50)	0.54
rs2289728×HBsAg	0.02	0.53	1.02 (0.36-2.86)	0.98
rs139394×Smoking habit	-0.61	0.59	0.54 (0.17-1.72)	0.30
rs139394×Alcohol intake	-0.71	0.61	0.49 (0.15-1.60)	0.24
rs139394×HBsAg	0.27	0.48	1.31 (0.51-3.37)	0.58

a: adjusted age, gender, smoking habits, alcohol intake, and HBsAg values by logistic regression and the independent effect of each SNP. Values of these covariates in logistic regression models were shown in Table S2.

**Table 5:** Results of SNP-SNP interaction analyses.

Factors	*β*	SE (*βi*)	*OR *(95% *CI*)^a^	*P-*value ^a^
rs2289728× rs7217395	0.60	0.51	1.82 (0.67-4.99)	0.24
rs2289728× rs2036316	0.50	0.56	1.66 (0.55-4.95)	0.37
rs2289728× rs3764374	-0.04	0.54	0. 96 (0.33-2.79)	0.95
rs2289728× rs1285251	-0.735	0.57	0. 48 (0.16-1.46)	0.20
rs2289728×rs7292074	0.28	0.52	1.33 (0.48-3.68)	0.59
rs2289728×rs710190	0.51	0.52	1.66 (0.61-4.56)	0.33
rs2289728×rs139394	-0.57	0.52	0.57 (0.21-1.56)	0.27
rs2289728×rs5750753	0.71	0.57	2.04 (0.67-6.18)	0.21
rs139394× rs7217395	-0.30	0.48	0. 74 (0.29-1.91)	0.54
rs139394× rs2036316	0.28	0.53	1.327 (0.47-3.68)	0.60
rs139394× rs3764374	0.18	0.49	1.20 (0.46-3.12)	0.71
rs139394× rs1285251	0.69	0.48	2.00 (0.79-5.09)	0.15
rs139394× rs7292074	-0.07	0.49	0.93 (0.36-2.42)	0.89
rs139394× rs710190	-1.12	0.53	**0.33 (0.12-0.91)**	**0.03**
rs139394×rs5750753	-0.27	0.63	0.77 (0.22-2.62)	0.67

Bold values indicate statistical significance.

a: adjusted age, gender, smoking habits, alcohol intake, and HBsAg values by logistic regression and the independent effect of each SNP. Values of these covariates in logistic regression models were shown in Table S3.

## Data Availability

The data used to support the findings of this study is available from the corresponding author upon request.

## Supplementary Materials

Supplementary Materials The supplementary material file contains the values of the covariates in logistic regression models of Tables 3, 4, and 5. Table S1: covariates in logistic regression models of associations between SNPs and HCC. Table S2: covariates in logistic regression models of gene-environment interaction analyses. Table S3: covariates in logistic regression models of SNP-SNP interaction analyses.
