# Supplementary material for: Single Nucleotide Polymorphisms of CBX4 and CBX7 Decrease the Risk of Hepatocellular Carcinoma
Source: Biomed Res Int. 2019 May 9;2019:6436825. doi: 10.1155/2019/6436825 (PMC6532305; doi:10.1155/2019/6436825)
Supplement: Supplementary Materials — The supplementary material file contains the values of the covariates in logistic regression models of Tables 3, 4, and 5. Table S1: covariates in logistic regression models of associations between SNPs and HCC. Table S2: covariates in logistic regression models of gene-environment interaction analyses. Table S3: covariates in logistic regression models of SNP-SNP interaction analyses. [file 6436825.f1.pdf]

Table S1: Covariates in logistic regression models of associations between SNPs and HCC

Factors	β	SE(βi)	*Wald*	OR(95% *CI*)[a]	*P*-value
rs7217395					
Age	0.02	0.01	3.74	1.02(1.00-1.04)	0.05
Gender	-0.62	0.30	4.19	0.54(0.30-0.97)	0.04
Smoking habit	0.76	0.36	4.42	2.13(1.05-4.31)	0.04
Alcohol intake	0.78	0.37	4.49	2.19(1.06-4.52)	0.03
HBsAg	3.61	0.25	204.95	37.06(22.60-60.77)	0.00
rs2036316					
Age	0.02	0.01	4.72	1.02(1.00-1.04)	0.03
Gender	-0.66	0.31	4.57	0.52(0.28-0.95)	0.03
Smoking habit	0.91	0.36	6.37	2.48(1.22-5.00)	0.01
Alcohol intake	0.64	0.37	3.07	1.90(0.93-3.90)	0.08
HBsAg	3.61	0.25	203.08	37.09(22.56-60.96)	0.00
rs3764374					
Age	0.02	0.01	4.35	1.02(1.00-1.04)	0.04
Gender	-0.49	0.30	2.63	0.61(0.34-1.11)	0.11
Smoking habit	0.74	0.36	4.14	2.10(1.03-4.27)	0.04
Alcohol intake	0.66	0.37	3.16	1.94(0.93-4.03)	0.08
HBsAg	3.58	0.26	192.82	35.95(21.68-59.66)	0.00
rs1285251					
Age	0.03	0.01	6.02	1.03(1.01-1.05)	0.01
Gender	-0.60	0.30	3.84	0.55(0.30-1.00)	0.05
Smoking habit	0.81	0.35	5.25	2.24(1.12-4.46)	0.02
Alcohol intake	0.71	0.36	3.87	2.03(1.00-4.11)	0.05
HBsAg	3.62	0.25	204.16	37.19(22.65-61.07)	0.00
rs2289728					
Age	0.02	0.01	4.48	1.02(1.00-1.04)	0.03
Gender	-0.62	0.30	4.24	0.54(0.30-0.97)	0.04
Smoking habit	0.86	0.35	5.92	2.37(1.18-4.74)	0.02
Alcohol intake	0.75	0.36	4.26	2.12(1.04-4.32)	0.04
HBsAg	3.63	0.25	206.56	37.61(22.93-61.68)	0.00
rs7292074					
Age	0.03	0.01	7.29	1.03(1.01-1.05)	0.01
Gender	-0.46	0.30	2.42	0.63(0.35-1.13)	0.12
Smoking habit	0.82	0.35	5.41	2.26(1.14-4.50)	0.02
Alcohol intake	0.73	0.36	4.06	2.07(1.02-4.20)	0.04
HBsAg	3.58	0.25	205.88	35.99(22.06-58.72)	0.00
rs710190					

Age	0.02	0.01	4.91	1.02(1.00-1.04)	0.03
Gender	-0.58	0.30	3.76	0.56(0.31-1.01)	0.05
Smoking habit	0.87	0.35	6.03	2.39(1.19-4.78)	0.01
Alcohol intake	0.73	0.36	4.03	2.07(1.02-4.22)	0.05
HBsAg	3.58	0.25	205.86	36.03(22.08-58.80)	0.00
rs139394					
Age	0.03	0.01	5.81	1.03(1.01-1.05)	0.02
Gender	-0.54	0.31	3.13	0.58(0.32-1.06)	0.08
Smoking habit	0.73	0.36	4.04	2.07(1.02-4.20)	0.04
Alcohol intake	0.78	0.37	4.49	2.19(1.06-4.51)	0.03
HBsAg	3.64	0.26	203.93	37.89(23.01-62.40)	0.00
rs5750753					
Age	0.02	0.01	5.45	1.02(1.00-1.04)	0.02
Gender	-0.53	0.29	3.30	0.59(0.33-1.04)	0.07
Smoking habit	0.85	0.35	5.93	2.33(1.18-4.60)	0.02
Alcohol intake	0.79	0.36	4.92	2.21(1.10-4.46)	0.03
HBsAg	3.47	0.24	203.23	32.25(20.01-52.00)	0.00

Table S2: Covariates in logistic regression models of gene-environment interaction analyses

Factors	*β*	SE(*β*i)	*Wald*	*OR*(95% *CI*)[a]	*P*-value
rs2289728×Smoking habit					
Age	0.02	0.01	4.46	1.02(1.00-1.04)	0.04
Gender	-0.62	0.30	4.20	0.54(0.30-0.97)	0.04
Smoking habit	0.40	1.18	0.12	1.49(0.15-15.10)	0.74
Alcohol intake	0.74	0.36	4.12	2.09(1.03-4.27)	0.04
HBsAg	3.63	0.25	206.21	37.73(22.99-61.93)	0.00
rs2289728×Alcohol intake					
Age	0.02	0.01	4.39	1.02(1.00-1.04)	0.04
Gender	-0.61	0.30	4.15	0.54(0.30-0.98)	0.04
Smoking habit	0.89	0.36	6.24	2.43(1.21-4.89)	0.01
Alcohol intake	1.45	1.21	1.44	4.25(0.40-45.13)	0.23
HBsAg	3.62	0.25	206.72	37.42(22.84-61.30)	0.00
rs2289728×HBsAg					
Age	0.02	0.01	4.42	1.02(1.00-1.04)	0.04
Gender	-0.61	0.30	4.16	0.54(0.30-0.98)	0.04
Smoking habit	0.87	0.35	6.00	2.38(1.19-4.76)	0.01
Alcohol intake	0.75	0.36	4.22	2.11(1.04-4.30)	0.04
HBsAg	3.60	0.94	14.80	36.58(5.84-228.99)	0.00

rs139394×Smoking habit					
Age	0.02	0.01	5.13	1.02(1.00-1.04)	0.02
Gender	-0.52	0.30	2.97	0.59(0.33-1.08)	0.09
Smoking habit	1.65	0.92	3.23	5.21(0.86-31.61)	0.07
Alcohol intake	0.79	0.37	4.62	2.21(1.07-4.55)	0.03
HBsAg	3.62	0.25	205.82	37.50(22.85-61.52)	0.00
rs139394×Alcohol intake					
Age	0.02	0.01	5.29	1.02(1.00-1.04)	0.02
Gender	-0.51	0.30	2.85	0.60(0.33-1.09)	0.09
Smoking habit	0.76	0.36	4.43	2.14(1.05-4.33)	0.04
Alcohol intake	1.87	1.01	3.45	6.47(0.90-46.44)	0.06
HBsAg	3.62	0.25	205.46	37.32(22.75-61.22)	0.00
rs139394×HBsAg					
Age	0.02	0.01	5.33	1.02(1.00-1.05)	0.02
Gender	-0.53	0.30	3.02	0.59(0.33-1.07)	0.08
Smoking habit	0.77	0.36	4.62	2.16(1.07-4.37)	0.03
Alcohol intake	0.78	0.37	4.49	2.19(1.06-4.51)	0.03
HBsAg	3.22	0.75	18.50	25.11(5.78-109.07)	0.00

Table S3: Covariates in logistic regression models of SNP-SNP interaction analyses

Factors	*β*	SE(*β*i)	*Wald*	*OR*(95% *CI*)[a]	*P*-value
rs2289728× rs7217395					
Age	0.02	0.01	3.49	1.02(1.00-1.04)	0.06
Gender	-0.71	0.31	5.32	0.49(0.27-0.90)	0.02
Smoking habit	0.87	0.36	5.73	2.39(1.17-4.87)	0.02
Alcohol intake	0.76	0.37	4.14	2.13(1.03-4.41)	0.04
HBsAg	3.65	0.26	200.31	38.57(23.26-63.97)	0.00
rs2289728× rs2036316					
Age	0.02	0.01	3.83	1.02(1.00-1.04)	0.05
Gender	-0.70	0.31	5.14	0.50(0.27-0.91)	0.02
Smoking habit	0.95	0.36	6.94	2.60(1.28-5.27)	0.01
Alcohol intake	0.73	0.37	3.90	2.08(1.01-4.28)	0.05
HBsAg	3.60	0.26	196.09	36.40(22.01-60.21)	0.00
rs2289728× rs3764374					
Age	0.02	0.01	3.49	1.02(1.00-1.04)	0.06
Gender	-0.60	0.31	3.89	0.55(0.30-1.00)	0.05
Smoking habit	0.85	0.37	5.30	2.35(1.14-4.85)	0.02
Alcohol intake	0.71	0.38	3.51	2.04(0.97-4.29)	0.06
HBsAg	3.56	0.26	187.62	35.08(21.09-58.37)	0.00

rs2289728× rs1285251					
Age	0.02	0.01	5.57	1.02(1.00-1.05)	0.02
Gender	-0.69	0.31	5.06	0.50(0.28-0.92)	0.03
Smoking habit	0.89	0.36	6.20	2.44(1.21-4.91)	0.01
Alcohol intake	0.74	0.36	4.11	2.09(1.03-4.28)	0.04
HBsAg	3.64	0.26	201.14	38.23(23.11-63.26)	0.00
rs2289728×rs7292074					
Age	0.02	0.01	5.50	1.02(1.00-1.05)	0.02
Gender	-0.61	0.30	4.05	0.55(0.30-0.98)	0.04
Smoking habit	0.88	0.35	6.13	2.40(1.20-4.81)	0.01
Alcohol intake	0.75	0.36	4.20	2.11(1.03-4.30)	0.04
HBsAg	3.61	0.26	201.46	37.09(22.52-61.08)	0.00
rs2289728×rs710190					
Age	0.02	0.01	4.69	1.02(1.00-1.04)	0.03
Gender	-0.64	0.30	4.51	0.53(0.29-0.95)	0.03
Smoking habit	0.93	0.36	6.72	2.52(1.25-5.08)	0.01
Alcohol intake	0.74	0.37	4.13	2.10(1.03-4.31)	0.04
HBsAg	3.60	0.26	198.90	36.55(22.17-60.27)	0.00
rs2289728×rs139394					
Age	0.02	0.01	4.04	1.02(1.00-1.04)	0.04
Gender	-0.63	0.31	4.21	0.53(0.29-0.97)	0.04
Smoking habit	0.85	0.36	5.45	2.34(1.15-4.77)	0.02
Alcohol intake	0.77	0.37	4.19	2.15(1.03-4.47)	0.04
HBsAg	3.63	0.26	198.40	37.64(22.72-62.36)	0.00
rs2289728×rs5750753					
Age	0.02	0.01	4.28	1.02(1.00-1.04)	0.04
Gender	-0.64	0.30	4.50	0.53(0.29-0.95)	0.03
Smoking habit	0.85	0.36	5.71	2.34(1.17-4.71)	0.02
Alcohol intake	0.80	0.37	4.68	2.23(1.08-4.59)	0.03
HBsAg	3.57	0.26	196.72	35.55(21.58-58.55)	0.00
rs139394× rs7217395					
Age	0.02	0.01	3.56	1.02(1.00-1.04)	0.06
Gender	-0.62	0.31	3.95	0.54(0.30-0.99)	0.05
Smoking habit	0.73	0.37	3.93	2.08(1.01-4.27)	0.05
Alcohol intake	0.83	0.38	4.82	2.30(1.09-4.83)	0.03
HBsAg	3.60	0.26	197.42	36.43(22.06-60.15)	0.00
rs139394× rs2036316					
Age	0.02	0.01	4.30	1.02(1.00-1.04)	0.04

Gender	-0.66	0.31	4.39	0.52(0.28-0.96)	0.04
Smoking habit	0.86	0.37	5.51	2.36(1.15-4.83)	0.02
Alcohol intake	0.75	0.38	4.02	2.12(1.02-4.43)	0.05
HBsAg	3.58	0.26	194.66	36.03(21.77-59.60)	0.00
rs139394× rs3764374					
Age	0.02	0.01	4.06	1.02(1.00-1.04)	0.04
Gender	-0.48	0.31	2.45	0.62(0.34-1.13)	0.12
Smoking habit	0.72	0.38	3.68	2.05(0.98-4.29)	0.06
Alcohol intake	0.75	0.39	3.78	2.11(0.99-4.50)	0.05
HBsAg	3.56	0.26	188.74	35.18(21.17-58.46)	0.00
rs139394× rs1285251					
Age	0.03	0.01	5.87	1.03(1.01-1.05)	0.02
Gender	-0.55	0.31	3.16	0.58(0.32-1.06)	0.08
Smoking habit	0.78	0.36	4.70	2.19(1.08-4.44)	0.03
Alcohol intake	0.79	0.37	4.52	2.20(1.06-4.56)	0.03
HBsAg	3.65	0.26	199.82	38.33(23.12-63.55)	0.00
rs139394× rs7292074					
Age	0.03	0.01	6.26	1.03(1.01-1.05)	0.01
Gender	-0.52	0.30	2.90	0.60(0.33-1.08)	0.09
Smoking habit	0.79	0.36	4.78	2.20(1.09-4.45)	0.03
Alcohol intake	0.81	0.37	4.76	2.25(1.09-4.64)	0.03
HBsAg	3.60	0.26	198.96	36.49(22.14-60.16)	0.00
rs139394× rs710190					
Age	0.02	0.01	4.58	1.02(1.00-1.04)	0.03
Gender	-0.55	0.31	3.22	0.58(0.32-1.05)	0.07
Smoking habit	0.78	0.37	4.58	2.18(1.07-4.46)	0.03
Alcohol intake	0.85	0.38	5.08	2.34(1.12-4.90)	0.02
HBsAg	3.58	0.26	197.31	35.90(21.78-59.16)	0.00
rs139394×rs5750753					
Age	0.02	0.01	4.93	1.02(1.00-1.04)	0.03
Gender	-0.54	0.31	3.19	0.58(0.32-1.06)	0.07
Smoking habit	0.78	0.36	4.58	2.18(1.07-4.43)	0.03
Alcohol intake	0.82	0.37	4.78	2.26(1.09-4.70)	0.03
HBsAg	3.56	0.25	197.11	34.99(21.30-57.47)	0.00
